# Exploration is dependent on reproductive state, not social state, in a cooperatively breeding bird

**DOI:** 10.1093/beheco/arw119

**Published:** 2016-08-04

**Authors:** Hannah A. Edwards, Hannah L. Dugdale, David S. Richardson, Jan Komdeur, Terry Burke

**Affiliations:** ^a^Department of Animal and Plant Sciences, University of Sheffield, Sheffield S10 2TN, UK,; ^b^School of Biology, The Faculty of Biological Sciences, University of Leeds, Leeds LS2 9JT, UK,; ^c^Behavioural Ecology and Physiological Group, Groningen Institute for Evolutionary Life Sciences, University of Groningen, PO Box 11103 9700 cc, Groningen, The Netherlands,; ^d^School of Biological Sciences, University of East Anglia, Norwich Research Park, Norwich, Norfolk NR4 7TJ, UK, and; ^e^Nature Seychelles, PO BOX 1310, Mahe, Republic of Seychelles

**Keywords:** cooperative breeder, personality, Seychelles warbler, reproductive state, social state, state-dependent.

## Abstract

Personality is an intriguing phenomenon in populations because it constrains behavioral flexibility. One theory suggests that personality could be generated and maintained if dependent on asset protection. It is predicted that trade-offs with fitness expectations and survival probability encourage consistent behavioral differences among individuals (personality). Although not mutually exclusive, the social niche specialization hypothesis suggests that a group of individuals that repeatedly interact will develop personality to avoid costly social conflict. The point at which behavioral consistency originates in the social niche hypothesis is still unclear, with predictions for development after a change in social status. In the facultative cooperatively breeding Seychelles warbler (*Acrocephalus sechellensis*), residing on Cousin Island, breeding vacancies are limited and this forces individuals into different social roles. We used this system to test whether reproductive and social state predicted among-individual differences in exploration. We had 2 predictions. First, that an individual’s start in life can predict personality, whereby young individuals with a good start to life (associated with early age reproduction and earlier onset survival senescence) are fast explorers, suggesting reproductive state-dependence. Second, that an individual’s social status can predict personality, whereby dominant individuals will be fast explorers, suggesting that the behavior is social state-dependent. Neither of the behaviors was associated with social state and social state did not affect behavioral consistency. However, novel object exploration was associated with a proxy of reproductive state. Our results provide further support for state being a mechanism for generating individual differences in behavior.

## Lay Summary

An individual’s circumstances or properties—also known as state—maybe a mechanism encouraging behavioral differences among individuals. We investigated whether exploration was associated with social state or reproductive state (i.e., insect abundance at year of birth) in a wild cooperative breeder. We found fast novel object exploration was associated with young individuals born into years of high insect abundance (i.e., low future reproductive state), suggesting this behavior is reproductive state-dependent.

## INTRODUCTION

The occurrence of consistent differences in behavior among individuals, known as animal personality (Sih et al. 2004; Réale et al. 2007), is an intriguing phenomenon considering that a flexible behavioral response should enable individuals to adapt to varying environments (Wolf et al. 2007; Réale et al. 2010). Personality can be highly heritable (Dochtermann et al. 2015) and can affect fitness (Smith and Blumstein 2008), but little is known about how it is maintained at the individual or population level (Bell 2007). To gain further evolutionary understanding as to why personality is generated and maintained, we require longitudinal studies of personality in the wild. This is because captive environments can alter an individual’s behavioral expression and longitudinal studies on free-living organisms in the natural environment are a way to circumvent this (Stamps and Groothuis 2010).

Circumstances or properties that alter the costs and benefits associated with behavior are known as states (Dall et al. 2004; Wolf et al. 2007; Biro and Stamps 2008; Dingemanse and Wolf 2010). States are inherently slow changing but can encourage long-term stability in behavior if associated in a positive feedback loop (Luttbeg and Sih 2010). Behavior dependent on asset protection predicts that individuals with a high future reproductive state (i.e., high assets) will be consistently slow explorers and risk averse (behaviors that are often positively correlated, e.g., Quinn et al. 2012), in order to prevent predation, compared with those that have a low future reproductive state (Dall et al. 2004; Stamps 2007; Wolf et al. 2007). Although more study is needed, a few empirical studies have found support for this prediction. Slow exploratory behavior was associated with increased survival probability and hence high future reproductive states in wild great tits (*Parus major*, Nicolaus et al. 2012) and reduced risk-taking behavior was exhibited by young individuals with high future reproductive states in gray mouse lemurs (*Microcebus murinus*, Dammhahn 2012).

A less explored avenue of research is the potential for personality to be dependent on social state. Reproductive state and social state are not mutually exclusive because social status is often associated with breeding benefits, although in some species subordinates do have opportunities to breed (Richardson et al. 2002). The social niche specialization hypothesis suggests that a group of individuals in a social group that repeatedly interact will benefit by developing social niches (Bergmüller and Taborsky 2010; Montiglio et al. 2013). Social niches, such as social status, cause individuals to behave differently by reducing social conflict and encouraging behavioral consistency through positive feedback mechanisms such as learning and costs incurred by changing social niches (Bergmüller and Taborsky 2010; Wolf and Weissing 2010). The relationship between social status and behavioral differences among individuals is still unclear (Gómez-Laplaza 2002; Fox et al. 2009). However, a few studies have shown that a dominant social status correlates with fast exploration and bold and aggressive behavior in a territorial context (Verbeek et al. 1996; Dingemanse and de Goede 2004; Favati et al. 2014). Also, in a cooperatively breeding system, immature individuals that showed lower levels of a subordinate helping behavior were aggressive and fast exploring (Bergmüller and Taborsky 2007).

Investigating how social state may affect individual differences in behavior among individuals does not give any insight into how it may affect within-individual consistency in a population (Dingemanse et al. 2010). For example, it is still unclear at what point behavioral consistency within individuals originates under the social niche hypothesis (Carter et al. 2014). It has been postulated that within-individual behavioral consistency could occur after a change in social status, termed the “transition” hypothesis (Carter et al. 2014). Further studies are needed, but in support of this theory, meerkat (*Suricata suricata*) subordinate females that later became dominant exhibited different cooperative personalities after social status change compared with those that remained subordinate (Carter et al. 2014). Also, male domestic fowl (*Gallus gallus*) showed increases in vigilance, activity, and exploration when changed from a subordinate to a dominant social position (Favati et al. 2014).

Facultative cooperative breeders, such as the Seychelles warbler (*Acrocephalus sechellensis*), provide an excellent system in which to test the state dependency of personality. In this species, individuals can forego reproduction to raise offspring that are not their own (Cockburn 1998). They are highly territorial, and the limited number of breeding vacancies in the Cousin Island population forces many individuals into a subordinate social status indefinitely or until there is a dominant breeding vacancy (Komdeur 1991). A subordinate social status can bear a cost through the loss of direct breeding benefits and reduced body condition when helping to rear young (Richardson et al. 2002; van de Crommenacker et al. 2011). These social states could consequently encourage behavioral differences among individuals through character displacement or trade-offs with future reproductive state (Bergmüller and Taborsky 2010). Furthermore, individuals born in years of high food availability (high insect abundance at year of birth) reproduce at an earlier age but have an earlier onset of survival senescence compared with those born into years of low food availability (Hammers et al. 2013). This good or bad start could influence an individual’s future reproductive state and generate behavioral differences among individuals (e.g., Dingemanse et al. 2002; Wolf et al. 2007).

In this study, we investigate whether exploration (exploration of a novel environment and exploration of a novel object) is associated with current social state or future reproductive state. In an attempt to tease these 2 states apart, we tested 2 predictions. First, that an individual’s start in life can predict personality, whereby young individuals that have a good start to life, and thus breed at an earlier age and have early onset survival senescence, will be faster explorers than old individuals that have a bad start to life, suggesting that personality is associated with reproductive state. Second, that an individual’s social state can predict personality, whereby dominant individuals are faster explorers than subordinates, suggesting that personality is associated with current social state (e.g., social conflict and aspects of the social niche environment such as resource holding potential). Furthermore, we will investigate whether social state affects within-individual behavioral consistency, thus providing support to the ‘‘transition’’ hypothesis.

## METHODS

### Ethics statement

Local ethical regulations and agreements were followed for fieldwork. Nature Seychelles permitted us to work on Cousin Island Nature Reserve. The Seychelles Department of Environment and the Seychelles Bureau of Standards authorized fieldwork and sampling.

### Study system and site

Seychelles warblers were monitored on the main study island of Cousin (0.29 km^2^; 04°20′S, 55°40′E) during the winter (January–February) and summer (June–September) breeding seasons in 2010–2015, where they have been monitored intensively since 1981 (Komdeur 1991). During this time, social status and group memberships were identified, individuals were ringed with a metal British Trust for Ornithology ring and color ringed if necessary, and blood sampled (for sexing and genotyping). The sex of each individual is determined using molecular sexing methods (Griffith et al. 2002). The population experiences virtually no immigration and emigration between surrounding islands (Komdeur et al. 2004, 2015), and there is a 0.92±0.02 probability of annually resighting in the first 2 years of life and 0.98±0.01 probability of annually resighting in adults (Brouwer et al. 2010). Subsequently, birds are presumed dead if not seen after 1 year. On average, annual survival probability in the first year of life is 0.61±0.09 and 0.84±0.04 for adult birds (Brouwer et al. 2006). The mean life span of an individual is 5.5 years, and a maximum life span of 17 years has been recorded (Komdeur 1991; Barrett et al. 2013).

To determine territory boundaries, breeding status, and to observe interactions with other warblers, dominant females were followed for a minimum of 15min on a weekly basis throughout each summer and winter season. A subordinate status was assigned to individual birds (>5 months old) that were consistently seen in a territory and interacted with group members, but did not engage in dominant pair behavior. Subordinates may or may not help defend territories and may or may not help to raise offspring (Komdeur 1991). Dominant status was assigned when a pair of individuals was observed in a territory over multiple weeks and the individuals within the pair stayed within close proximity of one another and had frequent vocal interactions. The age of the first breeding attempt has been shown to range from 1 to 8 years old, with 48% breeding in their first year (Hammers et al. 2013). The Seychelles warblers are insectivorous and take 98% of their food from the underside of leafs of predominately *Pisonia grandis*, *Morinda citrifolia*, and *Ficus* sp. (Komdeur 1991, 1994). Insect abundance was thus measured over 14 locations across the island during the main breeding season (Komdeur 1992). Using this data, we then averaged insect abundance over these 14 locations per year to get an estimate of annual variation in food availability (Spurgin LG, Bebbington KL, Fairfield EA, Komdeur J, Burke T, Dugdale HL, et al., unpublished data).

### Personality assays

Birds were caught in mist nets throughout the summer of 2010 and the winter and summer breeding seasons of 2012–2015 for exploration of the novel environment and of 2013–2015 for exploration of a novel object. Once a bird was caught in a mist net it was extracted, measured for morphometric traits, taken back to the field station, given 5min in a bird bag, assayed for personality, and then released back at its territory. Exploration of a novel environment was tested in an Oxygen 4 tent (L322 × W340 × H210cm, Gelert Ltd Wigan). The tent contained 3 artificial trees each with 2 branches 45cm long (one attached at 95cm and the other at the top of the trunk) and a trunk 148cm high (adapted from Verbeek et al. 1994). By observing through a small opening (15.24 cm long by 6.35 cm wide) in the gauze of the tent door, the number of flights, hops, and the total number of trees visited were recorded during a 5-min period. A flight denoted a transfer between branches on the same tree, between trees or between floor and tree, or any movement greater than a branch length that involved flapping of the wings. A hop was described as both feet off the ground with no wing flapping, either on the same branch or on the floor. The combined number of hops, flights, and trees visited was totaled to give a measure of exploration (Edwards et al. 2015).

Exploration of a novel object was then tested 2min after the exploration assay to allow for habituation to the novel environment of the tent (see acclimation test, Edwards et al. 2015). A novel pink toy attached to a tree branch (95cm long) was inserted and positioned in the center of the tent (adapted from Verbeek et al. 1994). For each bird, we included a control assay with the novel toy excluded to confirm that the behavioral reaction resulted from the novel toy and not the tree branch it was attached to (Edwards et al. 2015). Behavior scores (hops, flights, and trees visited in 5min) in the novel object assay were therefore used as a measure of exploration (Edwards et al. 2015).

Personality assays were collected on 312 individuals (1 measure = 175 birds; 2 = 96; 3 = 25; 4 = 8; 5 = 5; 6 = 3; female = 150, male = 166) for novel environment exploration and 177 individuals (1 measure = 120 birds; 2 = 52; 3 = 4; 4 = 1; female = 84, male = 96) for novel object exploration. Plots of both traits after repeat testing can be found in Supplementary Figure S1 and S2. Novel environment exploration and novel object exploration are repeatable in this study species (Edwards et al. 2015; Edwards et al. forthcoming).

### Statistical analyses

All statistical analyses were performed in R 3.0.2. (R Development Core Team 2013).

#### Social or reproductive state-dependence

Generalized linear mixed models using a Poisson error distribution with a log link were run in the package MCMCglmm 2.17 (Hadfield 2009). For all models, we specified an Inverse Wishart prior (*V* = 1, *n* = 0.2), the posterior distribution was sampled every 100 iterations, with a burn-in period of 3000 iterations and a run of 203000 iterations. Convergence was assessed by autocorrelation values (*r* < 0.1), visual inspection of time series plots of the model parameters and using the heidel.diag and geweke.diag functions. We ran 2 models, with the responses of exploration of a novel environment and exploration of a novel object. The fixed effects included variables known to influence personality: social status at testing (subordinate or dominant) and an interaction with insect abundance at year of birth (mean = 4.61, variance = 3.76), age (novel environment age range: 36–5687 days, novel object age range: 60–1432 days, e.g., Fisher et al. 2015) and an interaction with insect abundance at year of birth (both a proxy for an association with reproductive state), assay number (to control for habituation, e.g., Dingemanse et al. 2012), sex (e.g., Schuett and Dall 2009), and body mass (standardized for time of day). Age (days) was mean centered (Gelman and Hill 2006) and included as a linear term. Tent color was also included as a fixed effect in the novel environment exploration model because it was shown to have an effect in previous analyses (Edwards et al. forthcoming). The model also included observer identity and bird identity as random effects to account to repeat observations. To facilitate visualization of the interaction term between insect abundance and age, we subset insect abundance into good and bad years (defined as above and below the mean [4.61] insect abundance across all years) and used lm to calculate the smoothed regression in ggplot2 (Wickham 2009).

#### Behavioral consistency at social status transition

Hierarchical generalized linear models (HGLM; Cleasby and Nakagawa 2011; Cleasby et al. 2014) allow for individual/group differences in the residual variance to measure how an individual’s behavior changes when measured repeatedly, and hence its predictability. Therefore, only individuals with repeat personality measures were included in this analysis. We included assay number, age, and sex as fixed effects and fitted the difference of status between measures into the dispersion part of the standard Poisson HGLM using the package HGLM 2.0-11 (Ronnegard et al. 2010). The social status differences were grouped as 1) individuals that remained subordinate (novel environment exploration *n* = 49 [female = 26 and male = 23]; novel object exploration *n* = 15 [female = 9 and male = 6]); 2) individuals that remained dominant (novel environment exploration *n* = 74 [female = 29 and male = 45]; novel object exploration *n* = 31 [female = 13 and male = 18]); or 3) individuals that transitioned from subordinate to dominant social status between behavioral measures (novel environment exploration *n* = 36 [female = 17 and male = 19]; novel object exploration *n* = 11 [female = 3 and male = 8]). No individuals transitioned from dominant to subordinate status. Bird identity was also included as a random effect to control for repeat measures. To assess the effect of social status difference on the residual variance, we compared the fit of a model with and without the social status fixed effect in the dispersion part of the HGLM, using the conditional Akaike information criterion (AIC) values (Cleasby et al. 2014). The model with the smaller conditional AIC value and a difference greater than 7 was interpreted as a better fit (Burnham et al. 2011). Modeling variance often requires a large sample size (Martin et al. 2011; van de Pol 2012). To ensure the small sample size of the novel object exploration assay was not biasing our estimates, we ran a simulation analysis with a Poisson HGLM. We then changed the sample sizes (*n* = 11–500) to investigate the effect on 2 simulated models’ parameter estimates. Simulated sample sizes of 11 individuals per group (representative of our social status grouping) did not sufficiently bias the model parameters, suggesting our sample sizes were sufficient to detect an effect (see Supplementary Table S1).

## RESULTS

### Social or reproductive state-dependence

**Figure 1:**
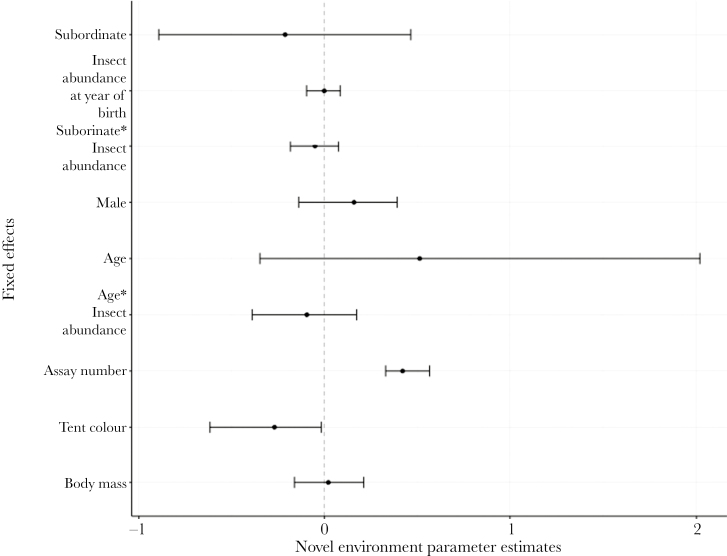
Posterior modes and associated 95% credible intervals of social status (contrast level = dominant, *N*: subordinates = 167, dominants = 145), insect abundance at year of birth and the interaction with social status, sex (contrast level = female, *N*: female = 149, male = 163), age and the interaction with insect abundance at year of birth, assay number*, tent color (contrast level = blue, *N*: blue = 244, green = 88)* and body mass in the novel environment exploration model. *Posterior modes whose 95% credible intervals do not overlap zero.

**Figure 2:**
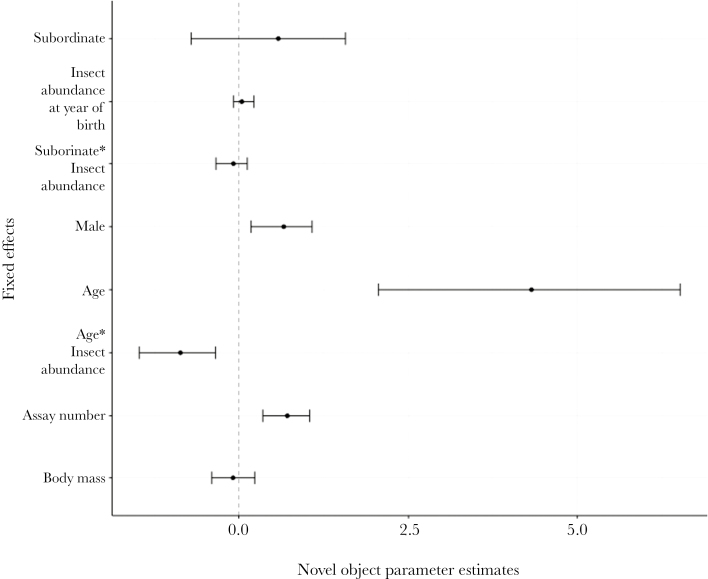
Posterior modes and associated 95% credible intervals of social status (contrast level = dominant, *N*: subordinate = 76, dominant = 101), insect abundance at year of birth and the interaction with social status, sex (contrast level = female, *N*: female = 81, male = 96)*, age and the interaction with insect abundance at year of birth*, assay number* and body mass in the novel object exploration model. *Posterior modes whose 95% credible intervals do not overlap zero.

**Figure 3:**
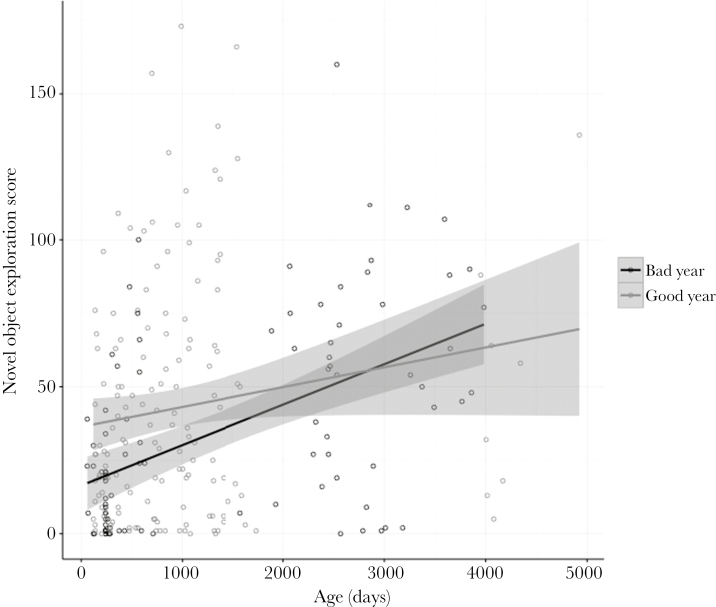
Novel object exploration scores of the Seychelles warblers of different ages (days), for individuals born into bad years (*n* = 30 individuals, black line and circles), and good years (*n* = 35 individuals, dark gray line and circles) of food abundance (defined as above or below the mean [4.61] insect abundance across all years). Insect abundance was a continuous effect in the model but is split into 2 categories on the graph to facilitate visualization of the interaction between insect abundance and age. 95% confidence intervals around the regression lines are in light gray.

Social state and insect abundance at year of birth were not associated with novel environment exploration (Figure 1 and Supplementary Table S2). Novel environment exploration instead increased with assay number. The marginal effects explained 0.19 (0.12–0.31) of the variance and the conditional effects explained 0.53 (0.27–0.73) of the variance in the novel environment exploration model. Social state was not associated with novel object exploration but with insect abundance at year of birth and the interaction with age (Figure 2 and Supplementary Table S3). There was a negative relationship between insect abundance and age, whereby young individuals born into years of high food abundance were associated with faster exploration of the novel object compared with individuals born into years of low food abundance (Figures 2 and 3). Males were faster explorers than females and novel object exploration also increased with assay number and age (Figure 2 and Supplementary Table S3). The marginal effects explained 0.22 (0.15–0.28) of the variance and the conditional effects explained 0.52 (0.39–0.62) of the variance in the novel object exploration model.

### Behavioral consistency at social status transition

For novel environment exploration, the standard Poisson model and the model with social status fixed in the dispersion were of similar fit (standard Poisson HGLM cAIC = 2864.63, h-likelihood = 2891.61 and modified dispersion HGLM cAIC = 2871.01, h-likelihood = 2896.68). Similarly, for novel object exploration, the 2 models were of similar fit (standard Poisson HGLM cAIC = 1137.75, h-likelihood = 1102.13 and modified dispersion HGLM cAIC = 1135.46, h-likelihood = 1105.94). Thus, there was no evidence for within-individual behavioral consistency being affected by social state.

## DISCUSSION

Asset protection could encourage consistent behavioral differences through trade-offs with future fitness expectations and survival probability (Wolf et al. 2007). The social niche specialization hypothesis further suggests that in a group where individuals repeatedly interact, consistent behavioral differences will develop in order to avoid costly social conflict (Bergmüller and Taborsky 2007; Laskowski and Pruitt 2014). In our study, we found that between-individual differences in exploration were not associated with social state and that within-individual behavioral consistency was unaffected by social state. However, insect abundance at year of birth, a proxy of reproductive state, and the interaction with age did predict novel object exploration.

Young individuals born in years of greater food abundance were associated with fast exploration of the novel object. This finding is in line with the asset protection theory that suggests individuals should adjust their risk-taking/exploratory behavior when there are trade-offs with future fitness expectations and survival probability (Wolf et al. 2007). This relationship has also been confirmed in empirical studies, where slow exploratory and reduced risk-taking behaviors are associated with individuals with high future reproductive states (Dammhahn 2012; Nicolaus et al. 2012). In the Seychelles warbler, greater food abundance at year of birth causes individuals to reproduce for the first time at an earlier age but results in earlier survival senescence (Hammers et al. 2013). Reproductive tactics are thus modified to suit environmental conditions to maintain survival (Hammers et al. 2013) and reproductive output is age dependent, with an initial increase followed by a decline in old age (Hammers et al. 2012). Young individuals born into good insect abundance years (low future reproductive states) may exhibit risky behavior, such as territory guarding and novel foraging, to ensure the success of current reproductive attempts. Long-term studies should look at how novel object exploration may be linked with aspects of the Seychelles warblers’ ecology, such as predator susceptibility or resource holding.

Novel object exploration was also associated with age, with older individuals exhibiting faster exploration. Previous studies have found that among-individual variance in personality traits increase later in life (Roberts and DelVecchio 2000; Fisher et al. 2015). It could be that older individuals have experienced greater environmental variation (novel prey or novel conspecifics) and are faster explorers than younger individuals. Furthermore, processes such as stimulus generalization (transfer of a response learned from one stimulus to a similar stimulus) in older individuals may encourage fast exploration. These 2 processes coupled together could result in age-related behavioral differences.

We also show that assay number affected between-individual differences in exploration. Differences in how individuals habituate to a novel environment are associated with individual differences in learning (Light et al. 2011) and fearfulness (File 2001). After repeat testing, individuals are thought to overcome fear and explore novel environments more superficially compared with previous experiences (Verbeek et al. 1994). This effect is particularly pronounced in slow explorers (Carere et al. 2005). Clearly, repeat testing is conflated with habituation, and therefore, assay number should be accounted for when repeatedly measuring traits that are associated with learning and fearfulness.

Novel object exploration also differed between the sexes, with males being faster explorers than females. The direction of among-individual differences in exploration between the sexes can vary, and it has been postulated that sexual selection may play a role in encouraging these differences (reviewed in Schuett et al. 2010). Exploratory behavior has been correlated with spatial response to territory intrusion, with fast explorers spending more time in proximity to the intruder in great tits (Snijders et al. 2015). In the Seychelles warbler, attack frequencies toward a simulated predator were higher in males, than in females (Veen et al. 2000). Exploration may therefore be associated with territorial defense, and if selected for in males would result in these sex differences.

Behavioral consistency of exploration was unaffected by social state, although this has been suggested as a mechanism for within-individual behavioral consistency in meerkats (Carter et al. 2014). In the Seychelles warbler, it could be that individuals decide on a life-history trajectory early in life and this is why social state had no effect (e.g., Bergmüller and Taborsky 2007). There are, however, some limitations to our study. We were unable to distinguish causation because we did not experimentally manipulate individuals and relied on natural changes in social status. Individuals could therefore have been predisposed to certain changes in social statuses caused by environmental, physiological or experiential factors. Also, behavioral variation within individuals was not captured over a lifetime (personality tested birds had an average age of 2.98 years, but Seychelles warblers have a mean life span of 5.5 years, Komdeur 1991). For example, subordinates that remained subordinate between personality measures could potentially transition to dominance at a later stage, and dominants that remained dominant may not have been assayed when subordinate. It is difficult to decipher whether the 3 social status groups (individuals that remained subordinate, individuals that remained dominant, or individuals that transitioned from subordinate to dominant social status between behavioral measures) were equally plastic or equally consistent, because variation within all the groups was the same. Both scenarios have their advantages for an individual. Behavioral plasticity can be adaptive (Sih et al. 2004; Kontiainen et al. 2009; Betini and Norris 2012) and allow individuals to display costly behaviors only when required. On the other hand, consistent behavior can allow individuals to specialize in different social niches and avoid costly social conflict (Bergmüller and Taborsky 2007).

## SUMMARY

We have shown that social state does not explain behavioral differences in exploration nor affect behavioral consistency. Instead we show that a proxy of reproductive state, sex, and age affect individual differences in novel object exploration, and repeat testing affects individual differences in novel environment exploration. Our results provide further support that exploration can be reproductive state-dependent and that this may be a mechanism for generating individual differences. We suggest that future work should look directly at survival probability as a mechanism for encouraging personality and sex-specific behavior such as territorial defense, which may encourage sex differences.

## SUPPLEMENTARY MATERIAL

Supplementary material can be found at http://www.beheco.oxfordjournals.org/

## FUNDING

This work was supported by a Natural Environment Research Council Studentship (X/007/001-15 to H.A.E.), a Natural Environment Research Council fellowship (NE/I021748/1 to H.L.D.), and 2 Schure Beijerinck Popping grants (SBP2013/46 to H.A.E. and SBP2012/26 to H.L.D.).

## Supplementary Material

Supplementary Data
