# Supplementary material for: Exploration is dependent on reproductive state, not social state, in a cooperatively breeding bird
Source: Behav Ecol. 2016 Aug 4;27(6):1889–96. doi: 10.1093/beheco/arw119 (PMC5181527; doi:10.1093/beheco/arw119)
Supplement: Supplementary Data [file supp_27_6_1889__index.html]

Exploration is dependent on reproductive state, not social state, in a cooperatively breeding bird — Exploration is dependent on reproductive state, not social state, in a cooperatively breeding bird — Supplementary Data 

# Exploration is dependent on reproductive state, not social state, in a cooperatively breeding bird

## Supplementary Data

Data files

- Supplementary Data - Supplementary Data
