# Supplementary material for: Exploration is dependent on reproductive state, not social state, in a cooperatively breeding bird
Source: Behav Ecol. 2016 Aug 4;27(6):1889–96. doi: 10.1093/beheco/arw119 (PMC5181527; doi:10.1093/beheco/arw119)
Supplement: Supplementary Data [file supp_arw119_Socialstate_Supp_BehavEco.docx]

**Supporting Information**

**Exploration is dependent on reproductive state, not social state, in a cooperatively breeding bird**

**Table S1:** A simulation analysis using a standard Poisson HGLM with one random effect of three groups and a varying number of individuals in each group. Simulated datasets were created with an intercept of 0 and variance of 1 and a variance of 0.17. The R package HGLM 2.0-11 was used to assess the effect of sample size changes on the model intercept and variance estimates.

| **Intercept 0, variance 1** | **Intercept mean** | **Variance mean** |
| --- | --- | --- |
| 3 groups, 11 observations per group | -0.07 | 1.18 |
| 3 groups, 35 observations per group | -0.01 | 0.97 |
| 3 groups, 50 observations per group | -0.09 | 1.25 |
| 3 groups, 100 observations per group | 0.05 | 1.14 |
| 3 groups, 200 observations per group | 0.04 | 1.12 |
| 3 groups, 500 observations per group | -0.04 | 1.11 |
| **Intercept 0, variance 0.17** | **Intercept mean** | **Variance mean** |
| 3 groups, 11 observations per group | -0.06 | 0.27 |
| 3 groups, 35 observations per group | -0.03 | 0.21 |
| 3 groups, 50 observations per group | -0.03 | 0.18 |
| 3 groups, 100 observations per group | -4e-3 | 0.19 |
| 3 groups, 200 observations per group | -0.03 | 0.19 |
| 3 groups, 500 observations per group | -0.04 | 0.16 |

**Table S2**: Estimates of the posterior distributions of the fixed effects in the Poisson novel environment exploration model for social status (contrast level = dominant; N: subordinates = 147, dominants = 169), insect abundance at year of birth and the interaction, sex (contrast level female; N: females = 150, males = 166), age and the interaction with insect abundance at year of birth, assay number, tent colour (contrast level = blue; N: blue = 288, green = 88) and body mass. Posterior means and associated 95% credible intervals are presented, with bold text indicating that an estimate differs from zero.

|  | **Posterior mode** | **Lower credible interval** | **Upper credible interval** |
| --- | --- | --- | --- |
| Subordinate | -0.21 | -0.89 | 0.46 |
| Insect abundance at year of birth | 0.00 | -0.10 | 0.08 |
| Subordinate* Insect abundance | -0.05 | -0.18 | 0.08 |
| Male | 0.16 | -0.14 | 0.39 |
| Age | 0.51 | -0.35 | 2.02 |
| Age* Insect abundance | -0.09 | -0.39 | 0.17 |
| Assay number | **0.42** | **0.33** | **0.56** |
| Tent colour | **-0.27** | **-0.61** | **-0.02** |
| Body mass | 0.02 | -0.16 | 0.21 |

**Table S3**: Estimates of the posterior distributions of the fixed effects in the Poisson novel object exploration model for social status (contrast level dominant, subordinate=81, dominant=99), insect abundance at year of birth and the interaction, sex (contrast level female, female=84, male=96), age and the interaction with insect abundance at year of birth, assay number and body mass. Posterior means and associated 95% credible intervals presented, bold text indicates significance, with bold text indicating that an estimate differs from zero.

|  | **Posterior mode** | **Lower credible interval** | **Upper credible interval** |
| --- | --- | --- | --- |
| Subordinate | 0.58 | -0.70 | 1.57 |
| Insect abundance at year of birth | 0.05 | -0.08 | 0.22 |
| Subordinate* Insect abundance | -0.08 | -0.34 | 0.13 |
| Male | **0.66** | **0.18** | **1.08** |
| Age | **4.33** | **2.06** | **6.51** |
| Age* Insect abundance | **-0.86** | **-1.48** | **-0.34** |
| Assay number | **0.72** | **0.35** | **1.04** |
| Body mass | -0.08 | -0.40 | 0.24 |

**Figures**

**Figure S1**: The change in novel environment exploration score with repeat testing. Lines represent all 312 individuals that were included in the main analyses.

**
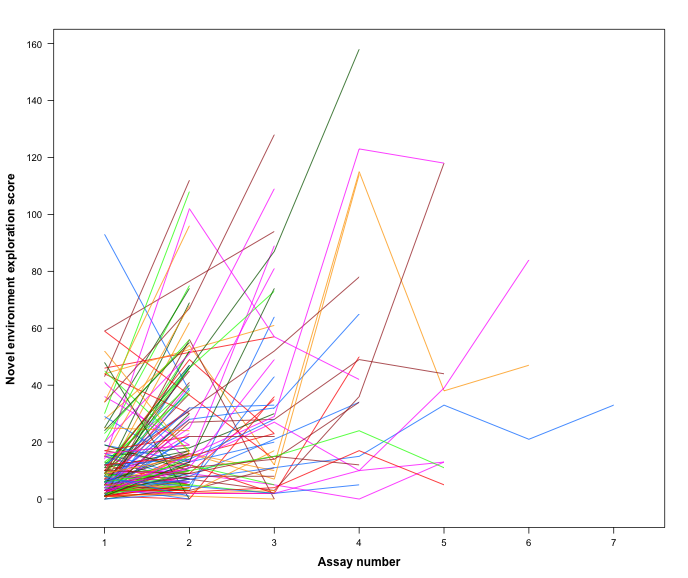
**

**Figure S2**: The change in novel object exploration score with repeat testing. Lines represent all 177 individuals that were included in the main analyses.

**
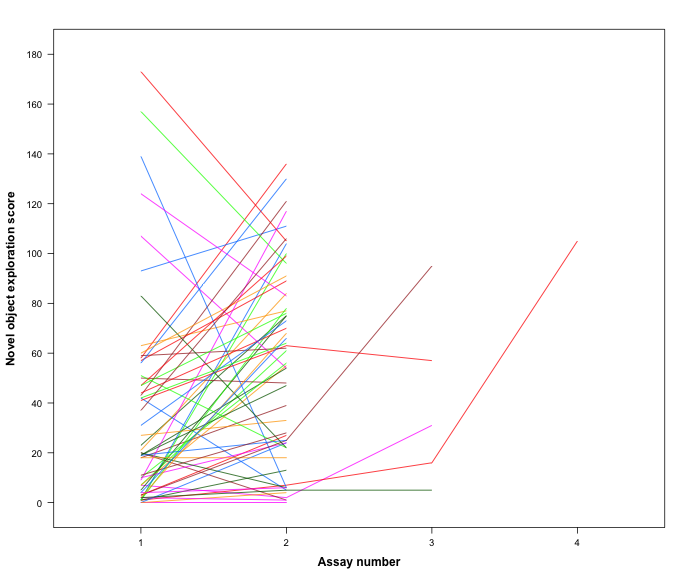
**
